# Mapping the functional expression of auxiliary subunits of K_Ca_1.1 in glioblastoma

**DOI:** 10.1038/s41598-022-26196-w

**Published:** 2022-12-20

**Authors:** Adam Feher, Zoltán Pethő, Tibor G. Szanto, Álmos Klekner, Gabor Tajti, Gyula Batta, Tibor Hortobágyi, Zoltan Varga, Albrecht Schwab, Gyorgy Panyi

**Affiliations:** 1 Department of Biophysics and Cell Biology, Faculty of Medicine, University Debrecen, Debrecen, Hungary; 2 Institute of Physiology II, University Münster, Münster, Germany; 3 Department of Neurosurgery, Faculty of Medicine, University Debrecen, Debrecen, Hungary; 4 Department of Genetics and Applied Microbiology, University Debrecen, Debrecen, Hungary; 5 Faculty of Medicine, Institute of Pathology, University of Szeged, Szeged, Hungary; 6 ELKH-DE Cerebrovascular and Neurodegenerative Research Group, Department of Neurology, Faculty of Medicine, University Debrecen, Debrecen, Hungary

**Keywords:** Biophysics, Cancer, Cell biology, Physiology

## Abstract

Glioblastoma (GBM) is the most aggressive glial tumor, where ion channels, including K_Ca_1.1, are candidates for new therapeutic options. Since the auxiliary subunits linked to K_Ca_1.1 in GBM are largely unknown we used electrophysiology combined with pharmacology and gene silencing to address the functional expression of K_Ca_1.1/*β* subunits complexes in both primary tumor cells and in the glioblastoma cell line U-87 MG. The pattern of the sensitivity (activation/inhibition) of the whole-cell currents to paxilline, lithocholic acid, arachidonic acid, and iberiotoxin; the presence of inactivation of the whole-cell current along with the loss of the outward rectification upon exposure to the reducing agent DTT collectively argue that K_Ca_1.1/β3 complex is expressed in U-87 MG. Similar results were found using human primary glioblastoma cells isolated from patient samples. Silencing the β3 subunit expression inhibited carbachol-induced Ca^2+^ transients in U-87 MG thereby indicating the role of the K_Ca_1.1/β3 in the Ca^2+^ signaling of glioblastoma cells. Functional expression of the K_Ca_1.1/β3 complex, on the other hand, lacks cell cycle dependence. We suggest that the K_Ca_1.1/β3 complex may have diagnostic and therapeutic potential in glioblastoma in the future.

## Introduction

Glioblastoma (GBM) is a significant health burden globally with frequent therapeutic failure and an abysmal long-term survival. In the clinical practice, classical chemotherapeutics, such as the alkylating agent temozolomide are combined with surgery and radiotherapy. Moreover, numerous clinical trials exist aiming to optimize chemotherapy with different treatment combinations, from which some show promising effects^1^. These studies are, however, still limited in number. Thus, new combination therapies are necessary to tackle the therapeutic challenge GBM imposes.

Ion channels are widely targeted in the therapy of various diseases and there is evidence that they may be promising targets in cancers, as well^2,3^. The Ca^2+^- and voltage dependent K^+^ channel K_Ca_1.1 (also known as BK_Ca_, Slo1 or MaxiK) is expressed as one of the major K^+^ channels in GBM cells^4,5^. Many of its functions are still unclear and disputed. Notably, the gBK (short for glioma BK) splice variant of K_Ca_1.1 is involved in the radiosensitivity of glioma cell lines^6^. However, the exact molecular mechanisms of these observations remain unclear to date. Even though radiosensitivity is altered by K_Ca_1.1, there is only scarce evidence whether channel modulation could potentiate GBM chemosensitivity^7^.

The pore-forming α subunit of K_Ca_1.1 is associated with auxiliary subunits. These subunits modify the biophysical characteristics of the channel and responsiveness to pharmacological modulators^8^. Moreover, the expression of a particular β subunit can be characteristic for certain pathological conditions. For example, we have showed earlier that CD44^+^ fibroblast-like synoviocytes isolated from rheumatoid arthritis patients display increased β3 subunit expression and augmented whole-cell K_Ca_1.1 currents^9^. The REpository of Molecular BRAin Neoplasia DaTa (REMBRANDT) glioma database indicates that the *KCNMB3* gene, encoding the K_Ca_1.1 β3 subunit, is expressed in a higher copy number in high-grade gliomas leading to a poorer prognosis compared to tumors expressing KCNMB2, the gene encoding the β2 subunit^10^. For comparison, the expression of the α subunit (*KCNMA1*) is upregulated only in ≈10% of GBM patients, and its overexpression does not correlate with overall patient survival^11^. However, there is no functional data to support the expression of K_Ca_1.1 α subunits in complex with β subunits in human glioblastoma cells or glioblastoma cell lines. To address this, we characterized the β subunits of K_Ca_1.1 in primary patient-derived GBM cells as well as in U-87 MG glioblastoma cell line using the combination of molecular biology, biophysics (electrophysiology) and pharmacology. Moreover, we investigated whether these auxiliary subunits regulate functional aspects and downstream effects of K_Ca_1.1.

## Results

### K_Ca_1.1 is a prominent K^+^ channel in the plasma membrane of glioblastoma cells

**Figure 1:**
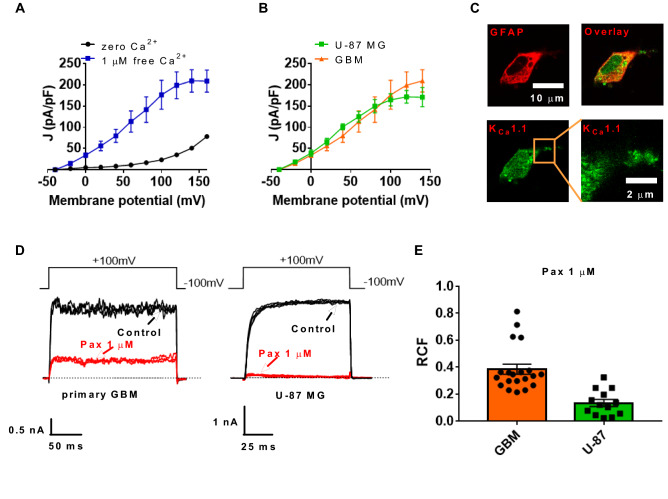
K_Ca_1.1 is a key K^+^ ion channel in glioblastoma cell membrane Current density–voltage relationship of whole-cell currents in primary glioblastoma cells. Current densities (J, pA/pF) at the indicated membrane potentials were calculated by dividing the peak current by the cell membrane capacitance. Data were obtained with intracellular solutions containing zero Ca^2+^ (black, n = 11) or 1 µM Ca^2+^ (blue, n = 5). (**B**) Current density–voltage relationship of whole-cell currents in primary glioblastoma cells (GBM, orange, n = 5) and in the U-87 MG glioblastoma cell line (U-87, green, n = 5). (**C**) Confocal microscopic images of a primary GBM cell. GFAP (top left) was labeled with anti-mouse Alexa 555, the K_Ca_1.1 alpha subunit was labeled with anti-rabbit Alexa 647, the overlay of the two images is in the top right. The bottom right panel depicts the punctate staining pattern of K_Ca_1.1 at higher magnification. (**D**) Representative whole-cell current traces recorded in a primary glioblastoma cell (GBM, left) and in a U-87 MG cell (U-87 MG, right). Currents were evoked by repeated depolarizations to + 100 mV from a holding potential of -100 mV (see voltage pulse above the raw current traces) every 15 s in control extracellular solution (black) and upon reaching equilibrium block in the presence of 1 µM paxilline (red). The pipette filling solution contained 1 µM Ca^2+^ concentration. (**E**) Remaining current fractions (RCF = I/I_0_, where I_0_ and I are the peak currents in the absence and in the presence of the inhibitor, respectively) of the outward currents in primary glioblastoma (GBM, orange bar) and U-87 MG cells (green bar) in the presence of 1 µM paxilline. Black symbols indicate RCF values obtained in individual cells. Throughout the figure, data points, bars and error bars are mean ± SEM for the indicated number of experiments.

First, we validated the functional expression of the K_Ca_1.1 in the membrane of GBM cells using the whole-cell patch-clamp technique. We expressed the currents as current density (J=pA/pF) where currents were normalized to the cell membrane capacitance to obtain a cell-size independent parameter. As seen in Fig. 1A, the voltage-gated K^+^ current density in primary GBM cells is markedly increased in the presence of 1 µM intracellular Ca^2+^ (N=3, n=5) compared to Ca^2+^−free intracellular solution (N=5, n=11) which is characteristic of K_Ca_1.1 channels (−40 mV, p=0.52; −20 mV, *p*=0.0004; 0 mV and above: *p*<0.0001, Student’s *t* tests). We also observed another hallmark of the K_Ca_1.1 channel^12^ that currents activate at much more negative membrane potentials in the presence of intracellular Ca^2+^. K^+^ currents of the glioblastoma cell line U-87 MG (N=3, n=5) have a similar current–voltage (I–V) relationship as in primary GBM cells (Fig. 1B) (N=3, n=11); which confirms the suitability of the cell line as a model to study K_Ca_1.1 in GBM. Also, whole-cell currents of primary GBM and U-87 MG cells are potently and irreversibly inhibited by applying 1 µM of the small-molecule blocker paxilline (Pax) (Fig. 1D). The remaining current fractions (RCF=I/I_0_, where I_0_ and I are the peak currents in the absence and in the presence of the inhibitor, respectively) in the presence of 1 µM paxilline were RCF=0.38 ± 0.04, n=20, and RCF=0.13 ± 0.02, n=13 for GBM and U-87 MG cells, respectively (Fig. 1E). Using immunofluorescence labels against K_Ca_1.1, we also detected a punctate membrane staining^13,14^ on the membrane of GFAP positive GBM cells (Fig. 1C). These results support previous reports that K_Ca_1.1 functions as a major K^+^ channel in GBM^4,5,15,16^.

### β3 is the main auxiliary subunit associated with the K_Ca_1.1 channel in glioblastoma

Auxiliary subunits of K_Ca_1.1 are known to alter the biophysical characteristics of the channel^8^. However, only very limited information is available on β subunit expression in GBM. Thus, we aimed at demonstrating the expression of β subunits using molecular biology and confirming the association of K_Ca_1.1 to its auxiliary β subunits with the combination of biophysical, pharmacological methods and genetic modulations.

**Figure 2:**
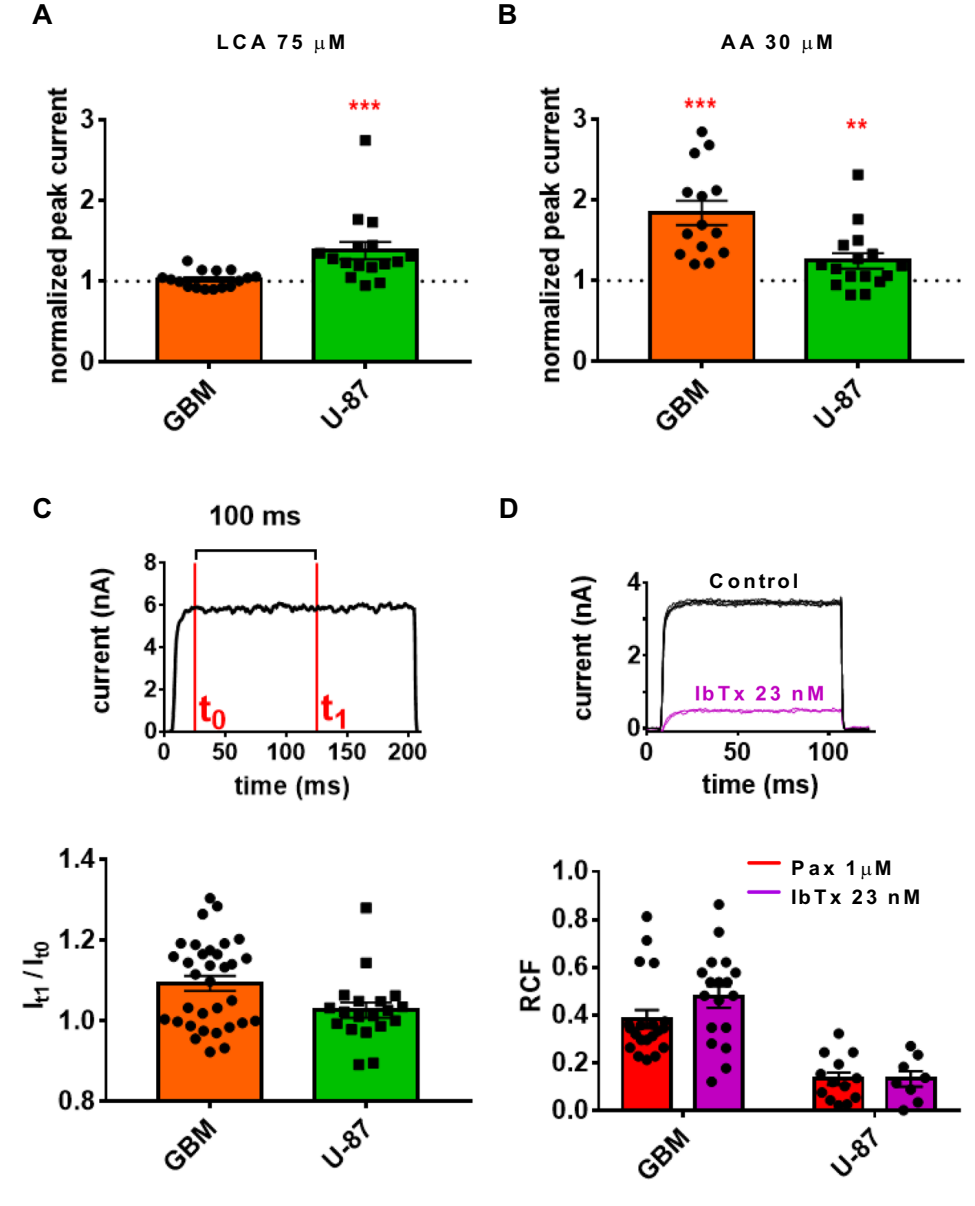
Pharmacological modulators affect β subunit-associated K_Ca_1.1 channels in glioblastoma. (**A**) The effect of lithocholic acid (LCA, 75 µM) on primary GBM (orange bar, n = 16) and U-87 MG cells (green bar, n = 16). The current recorded during LCA application was normalized to the current recorded in control solution (dotted line). (**B**) The effect of arachidonic acid (AA, 30 µM) on primary GBM (orange bar, n = 14) and U-87 MG (green bar n = 16) cells. The current recorded during AA application was normalized to the current recorded in control solution (dotted line). (**C**) Channel inactivation in a 100 ms time interval was determined by the current ratio I_t1_/I_t0_, where t_0_ was the current amplitude at the beginning, and t_1_ was the current amplitude at the end of the 100 ms time interval for primary GBM cells (orange bar, n = 33) and for U-87 cells (green bar, n = 19). The pipette filling solution contained 1 µM Ca^2+^ concentration. (**D**) The effect of iberiotoxin (IbTx, 23 nM, purple bars, for GBM n = 18, for U-87 n = 8) and paxilline (Pax, 1 µM, red bars, for GBM n = 20, for U-87 n = 13) on the K_Ca_1.1 current in primary GBM and in U-87 cells. Black symbols indicate remaining current fraction (RCF) values obtained in individual cells. RCF is defined in the text and in the legend of Fig. 1. Currents in panels C and D were evoked by depolarizations to + 100 mV, the pipette filling solution contained 1 µM Ca^2+^ concentration. Data in the bar graphs represent mean ± SEM, **p < 0.01, ***p < 0.001.

First, we aimed to determine the functional expression of the β subunits in primary GBM cells and in the U-87 MG cell line using the patch-clamp technique (Fig. 2). Both lithocholic acid (LCA) and arachidonic acid (AA) activate K_Ca_1.1 channels associated with the β1 subunit^17–19^, whereas AA also increases the current when K_Ca_1.1 is assembled with the β2 or β3 subunits^20^. In primary GBM cells, AA approximately doubles whole-cell K_Ca_1.1 currents compared to control (Fig. 2B, 1.84-fold increase, n=14, *p*=0.001, two-tailed Wilcoxon test), whereas LCA does not induce an increase of the K_Ca_1.1 current (Fig. 2A, 1.02-fold increase, n=16, *p*=0.63, two-tailed Wilcoxon test). In contrast, in U-87 MG cells, application of LCA and AA elicit a similar increase in the outward current compared to control (on average, 1.38-fold (n=16, *p*=0.0002) and 1.24-fold (n=16, *p*=0.008) current increase, respectively, two-tailed Wilcoxon test, Fig. 2A and 2B). K_Ca_1.1 channels co-expressed with β2 subunits inactivate with an inactivation time constant of around ~20 ms^21^ upon strong depolarization especially when the intracellular free Ca^2^^+^ concentration is high (10 µM). At low cytosolic Ca^2+^ concentrations and +100 mV depolarization, as in our study, the current would still significantly inactivate if β2 subunits are in complex with K_Ca_1.1α^22,23^. Data in Fig. 2C do not support the co-expression of the β2 subunits with K_Ca_1.1: whole-cell currents recorded from U-87 MG or primary GBM cells completely lack inactivation within 100 ms after the activation of the current. It is also known that K_Ca_1.1 channels co-expressed with β4 subunits are resistant against the peptide inhibitor iberiotoxin (IbTx)^24^. We tested the inhibition of the whole cell currents in primary GBM and U-87 MG cells at 23 nM IbTx concentration which is ~2–10-fold the IC_50_ for K_Ca_1.1 inhibition^25^ and compared the RCF to that determined using 1 µM paxilline, which is expected to block fully the KCa1.1 current when applied at negative holding potentials (IC_50_~12 nM). The use of ~ μM paxilline to define the K_Ca_1.1 current component of whole-cell currents in native cells is a commonly used strategy^26,27^. Fig. 2D shows that in case of primary GBM the RCF values are similar upon 23 nM IbTx (RCF = 0.47 ± 0.04 (n=18)) and 1 µM paxilline (RCF=0.38 ± 0.04, n=20, *p*=0.12, Student’s *t* tests) application. Similarly, the RCF values were statistically the same for the inhibition of the whole-cell currents in U-87 MG by 23 nM IbTx (RCF= 0.13 ± 0.03 (n=8) and 1 µM paxilline (RCF=0.13 ± 0.02, n=13 for, *p*=0.99, Student's *t* tests) (Fig. 2D). The similar extent of current inhibition for the peptide and non-peptide blocker precludes the presence of K_Ca_1.1 β4 subunits in the channel complex. In summary, the effect of pharmacological modulators on the β subunit-associated channels is consistent with the presence of the β1, β2, and β3 subunits in the K_Ca_1.1 complex, whereas the existence of the β4 subunit/K_Ca_1.1 complex in the cell membrane seems to be highly unlikely. This, combined with the lack of current inactivation (under conditions above) reduces the β subunit repertoire to β1 and β3 to combine with K_Ca_1.1 in GBM or U-87 MG.

**Figure 3:**
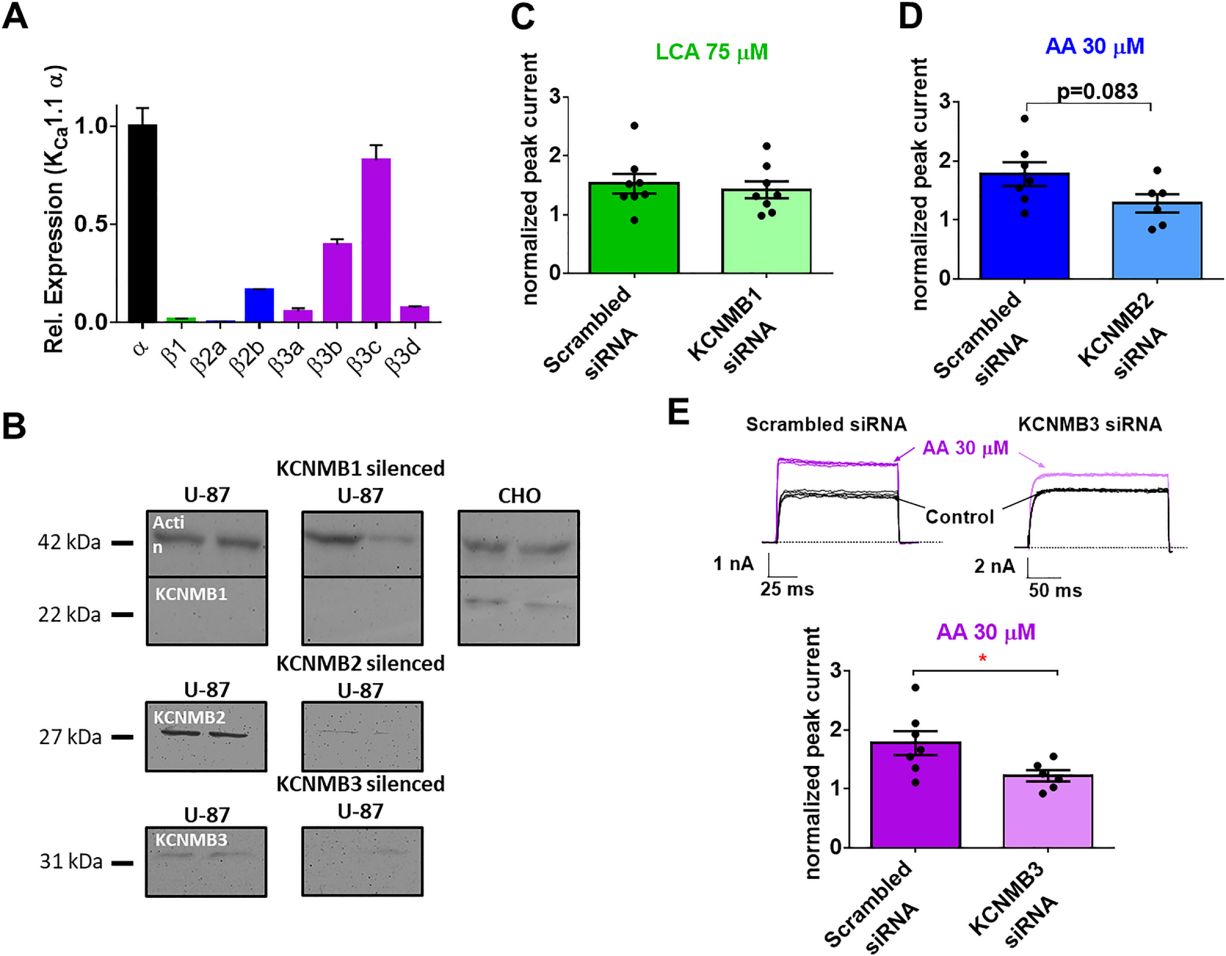
PCR, pharmacology and gene silencing is consistent with the expression of the K_Ca_1.1 β3 subunit in U-87 MG cells. (**A**) Results of qPCR experiments assessing the relative expression of the β subunits in the U-87 MG cell line compared to the pore-forming α subunit (black bar). β1, β2 and β3 mRNA levels divided by the expression of the K_Ca_1.1 channel α-subunit are represented with green, blue and purple bars, respectively (N = 3, n = 3). (**B**) Western blot of the untreated and gene-silenced U-87 MG cells. The populations were tested in duplicate, the left two lanes are the untreated U-87 MG cells and the right two lanes are untreated (non-transfected and not gene silenced) CHO cells. The middle lane pairs are for the *KCNMB1* (β1)*, KCNMB2* (β2) and *KCNMB3* (β3) silenced populations of U-87 MG, from top to bottom, respectively. The thick band in the top box at 42 kDa corresponds to actin and the 22 kDa marker to the β1 subunit (*KCNMB*1 gene product). In the second box (middle) the β2 band (27 kDa marker, KCNMB2 gene product), and in the third box (bottom) the β3 band (KCNMB3 gene product) are shown (31 kDa marker). (C to E) Pharmacological studies after gene silencing (N_Silence_ = 3). Whole-cell currents were recorded as in Fig. 1D, peak currents were measured and normalized peak currents were calculated as in Fig. 2A and B. (**C**) Effect of 75 μM LCA on control scrambled siRNA (dark green bar, n = 8) and on β1 silenced (*KCNMB1* siRNA transfected, light green bar, n = 8) U-87 MG cells. (**D**) Effect of 30 μM AA on the β2 silenced (*KCNMB2* siRNA transfected, light blue bar, n = 6), and (**E**) on the β3 silenced (*KCNMB3* siRNA transfected, light purple bar, n = 6), compared to the scrambled siRNA transfected groups (dark blue and dark purple bars respectively, n = 7). Insets in E) show the raw currents obtained in a scrambled RNA (left) and in a *KCNMB3* siRNA treated cell before (Control, black current traces) and after the application of 30 μM AA (purple current traces). Voltage protocols and solutions are as in Fig. 1D. Throughout the figure, bars and error bars indicate mean ± SEM for the indicated number of experiments (black symbols), *p < 0.05.

Next, we aimed at supporting the functional data using molecular biology techniques. As indicated in Fig. 3A, several K_Ca_1.1 auxiliary subunit mRNAs are expressed in the U- 87 MG cell line. Using RT-qPCR, we found that β1 and β2 subunits are expressed at low levels in both the primary tumor and U-87 MG cells, whereas the β3 subunit shows the highest expression (Supp. Fig. 1 for primary GBM).

As small-molecule pharmacological modulators such as LCA and AA activate multiple ion channels and signaling pathways^28,29^, we applied genetic modulation of U-87 MG cells using siRNAs targeting different K_Ca_1.1 β subunits to correlate β subunit expression and LCA/AA effects. Western blots in Fig. 3B demonstrate that the visible bands identified by anti-K_Ca_1.1 β2 (KCNMB2) or β3 (KCNMB1) antibodies cannot be detected after the application of the corresponding siRNAs for 48 h (Fig. 3B). As the anti-K_Ca_1.1 β1 (KCNMB1) antibody failed to identify any protein bands on the gel we applied this antibody to Chinese hamster ovary (CHO) cells were a band consistent with the presence of β1 (*KCNMB1*) expression was identified (mRNA of *KCNMB1* was reported previously in CHO cells^30^).

The functional consequence of β subunit silencing was tested using the pharmacological modulators LCA and AA. Lithocholic acid has a similar effect on whole-cell currents after K_Ca_1.1 β1 silencing compared to the scrambled siRNA control (*p*=0.63, Student’s *t*-test, N_silence_=3) (Fig. 3C). In contrast, both K_Ca_1.1 β2 and β3 siRNA-treatment decreases the response of U-87 MG cell to arachidonic acid compared to scrambled RNA silencing (*p*=0.083 and *p*=0.038, respectively) (Fig. 3D and 3E). Silencing of the β3 auxiliary subunit did not affect the sensitivity of the current to paxilline (Supp. Fig. 2). Taken together the higher sensitivity of the β3 silencing on the AA response (Fig. 3E) and the larger expression of the β3 RNA compared to other auxiliary subunits (Fig. 3A) suggest that K_Ca_1.1 β3 may be the main auxiliary subunit associated with K_Ca_1.1 in the membrane of glioblastoma cells.

**Figure 4:**
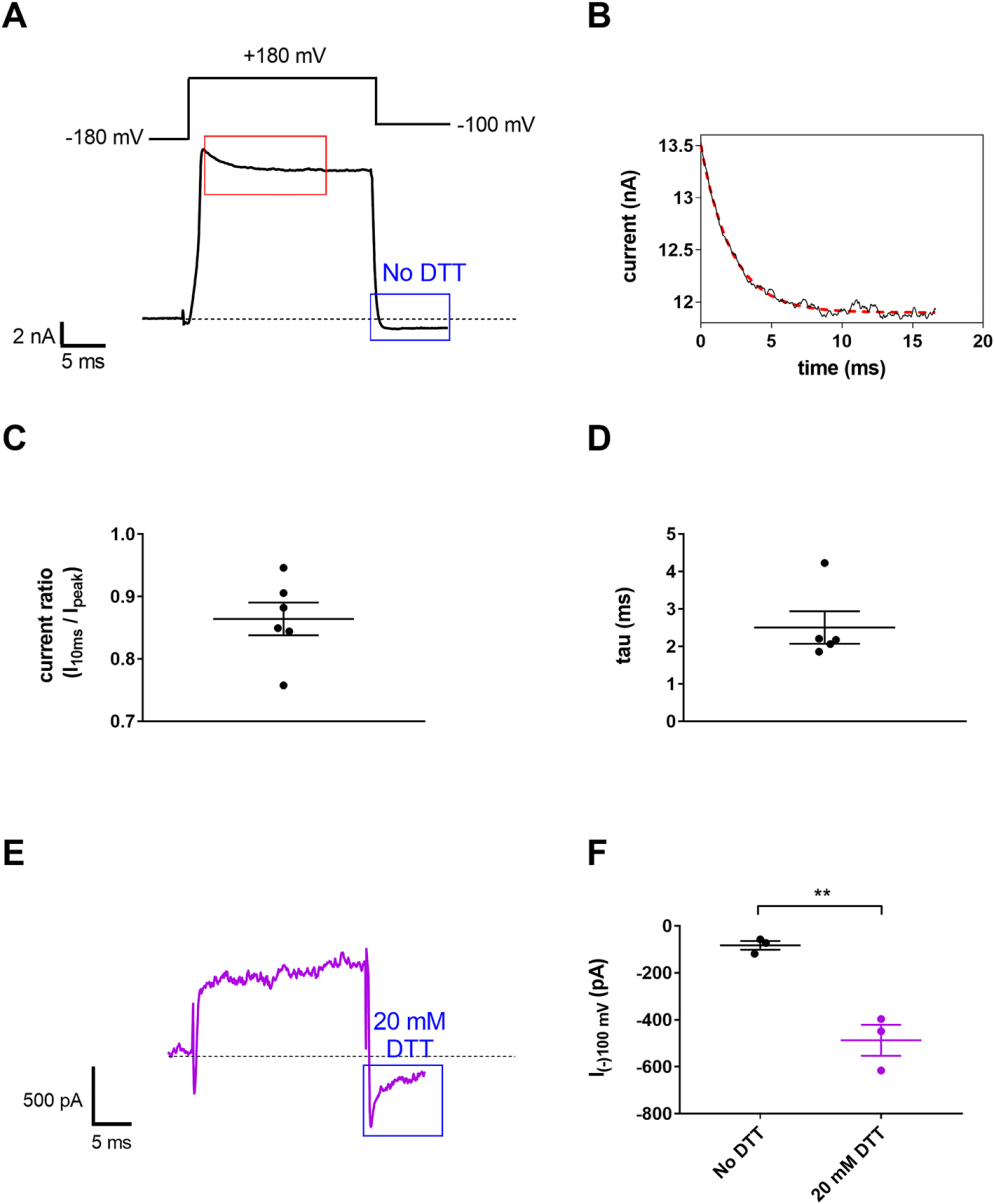
K_Ca_1.1 β3 subunit is functionally expressed in U-87 MG cells (**A**) Representative current trace recorded in a U-87 MG cell using the voltage protocol shown on the top and 10 µM free Ca^2+^ in the pipette filling solution. The red rectangle highlights current inactivation whereas the blue rectangle marks the current at − 100 mV in the absence of DTT. (**B**) Inactivation of the current (red rectangle in panel A) at an increased resolution. Continuous black line is the measured current, dashed red line is the fitted trace using a single exponential decay model. (**C**) The current ratio I_10ms_/I_peak_ denotes the ratio of the current at 10 ms following the start of the depolarization over the peak current. (**D**) Scatter plot of the inactivation time constants (tau) determined from fitting single-exponential decaying function to the currents. (**E**) Instantaneous (tail) current (blue rectangle) in a U-87 MG cell treated with 20 mM DTT for 20 min. The current was recorded using the pulse protocol in panel A. (**F**) Peak currents at − 100 mV for control U-87 MG cells in the absence of DTT (No DTT, black dots) and following 20 min application of 20 mM DTT (20 mM DTT, purple dots). Throughout the figure dots represent individual measurements horizontal line and error bars indicate mean ± SEM. * *p* < 0.05.

To ascertain the functional presence of the K_Ca_1.1 β3 auxiliary subunit in GBM cells, we performed biophysical studies with specialized patch-clamp protocols^31,32^. We increased the intracellular free Ca^2^^+^ concentration to 10 µM and depolarized the membrane to a more positive test potential (+ 180 mV). Under such conditions, K_Ca_1.1 channels associated to β3 subunits would be more likely to undergo a rapid and incomplete inactivation^31^. Indeed, as depicted in Fig. 4, the inactivation of the whole-cell current of U-87 MG cells follows this phenotype, as the current inactivation is incomplete, the I_10ms_/I_peak_ ratio, corresponding to the current at 10 ms following the start of the depolarization (I_10ms_) over the peak current (I_peak_), is decreased to 0.86 ± 0.03 (n = 6; Fig. 4C). Moreover, the inactivation kinetics is very rapid, the inactivation time constant (tau) is characteristically short (2.5 ± 0.43 ms, n = 5; Fig. 4D) for the KCa1.1/β3 complex under these experimental conditions^31^. Association of K_Ca_1.1 α subunits with β3 confers sensitivity of the complex to the reducing agent dithiothreitol (DTT)^32^. The outward rectification of the complex is abolished upon exposure to DTT as a consequence of the disruption of the disulfide links between the extracellular parts of the β3 subunits^21^. We exploited this phenomenon and showed that DTT treatment resulted in the appearance of the instantaneous current (i.e. tail current) when returning to a negative membrane potential (Fig. 4E and Supp. Fig. 3), whereas the tail current is absent without DTT application (Fig. 4A and Supp. Fig. 3). The peak currents at -100 mV were −487 ± 66 pA (n = 3) and 82 ± 18 pA (n = 3) respectively (Fig. 4F , *p* = 0.004, Student's *t* tests). The deactivation time constant of the tail current in the presence of 20 mM DTT was 1.68 ± 0.07 ms (n = 3), whereas in the absence of DTT the current at -100 mV does not deactivate within a 20 ms time period. These findings claim that β3 subunits are functionally present in the glioma cell membrane.

### K_Ca_1.1 β3 is involved in U-87 MG Ca^2+^ signaling but shows no cell cycle dependence

**Figure 5:**
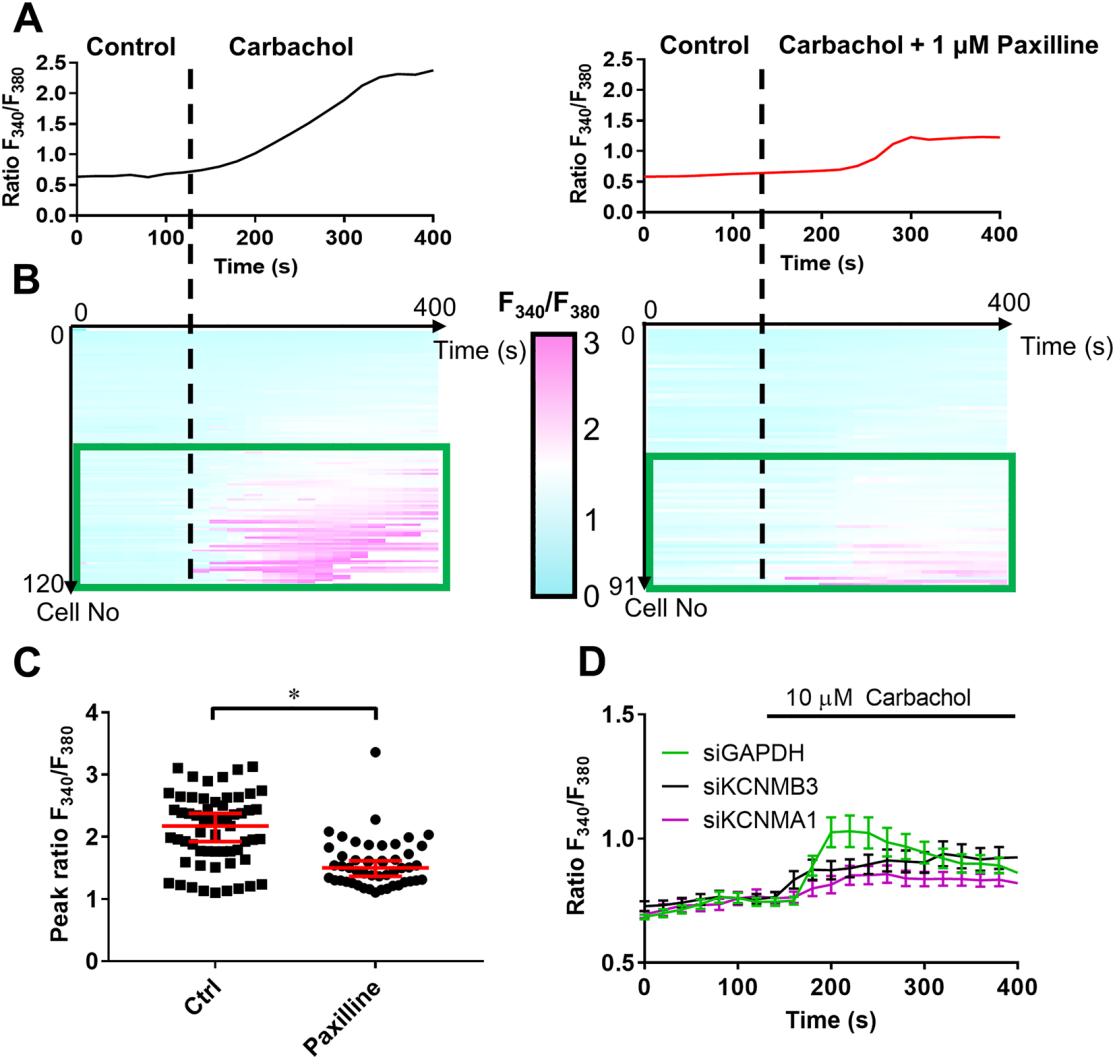
K_Ca_1.1 β3 is involved in the Ca^2+^ response of U87-MG cells (**A**) Representative intracellular Ca^2+^ measurements, F_340_/F_380_ ratio reports the intracellular Ca^2+^ concentration. Vertical dashed line indicates the beginning of carbachol (10 µM, left, black) or 10 µM carbachol + 1 µM paxilline (right, red) superfusion after a 2 min superfusion with control Ringer's solution (**B**) Heat map depicting the F_340_/F_380_ ratio response as a function of time for each individual cell measured. Left: 10 µM carbachol (N = 5, n = 120); right: 10 µM carbachol + 1 µM paxilline (N = 5, n = 91). Pseudocolor code shows increasing Ca^2+^ concentrations with more magenta-toned color. Green rectangle confines cells showing > 20% increase in F_340_/F_380_ ratio after carbachol superfusion. (**C**) Peak of the 10 µM carbachol-induced F_340_/F_380_ ratio (see A and B for details) of control superfused cells (n = 59, N = 3) and 1 µM paxilline-treated cells (n = 49, N = 3). (**D**) Carbachol-elicited intracellular Ca^2+^ response of cells after silencing of *GAPDH* (green, siGAPDH, N = 3, n = 39, F_340_/F_380_ = 1.1 ± 0.06), K_Ca_1.1 (purple, siKCNMA1, N = 3, n = 51, F_340_/F_380_ = 0.8 ± 0.04) and K_Ca_1.1 β3 (black, siKCNMB3, N = 3, n = 30, F_340_/F_380_ = 0.9 ± 0.04). In C) and D) data are mean ± SEM for the indicated number of experiments, *p < 0.05.

**Figure 6:**
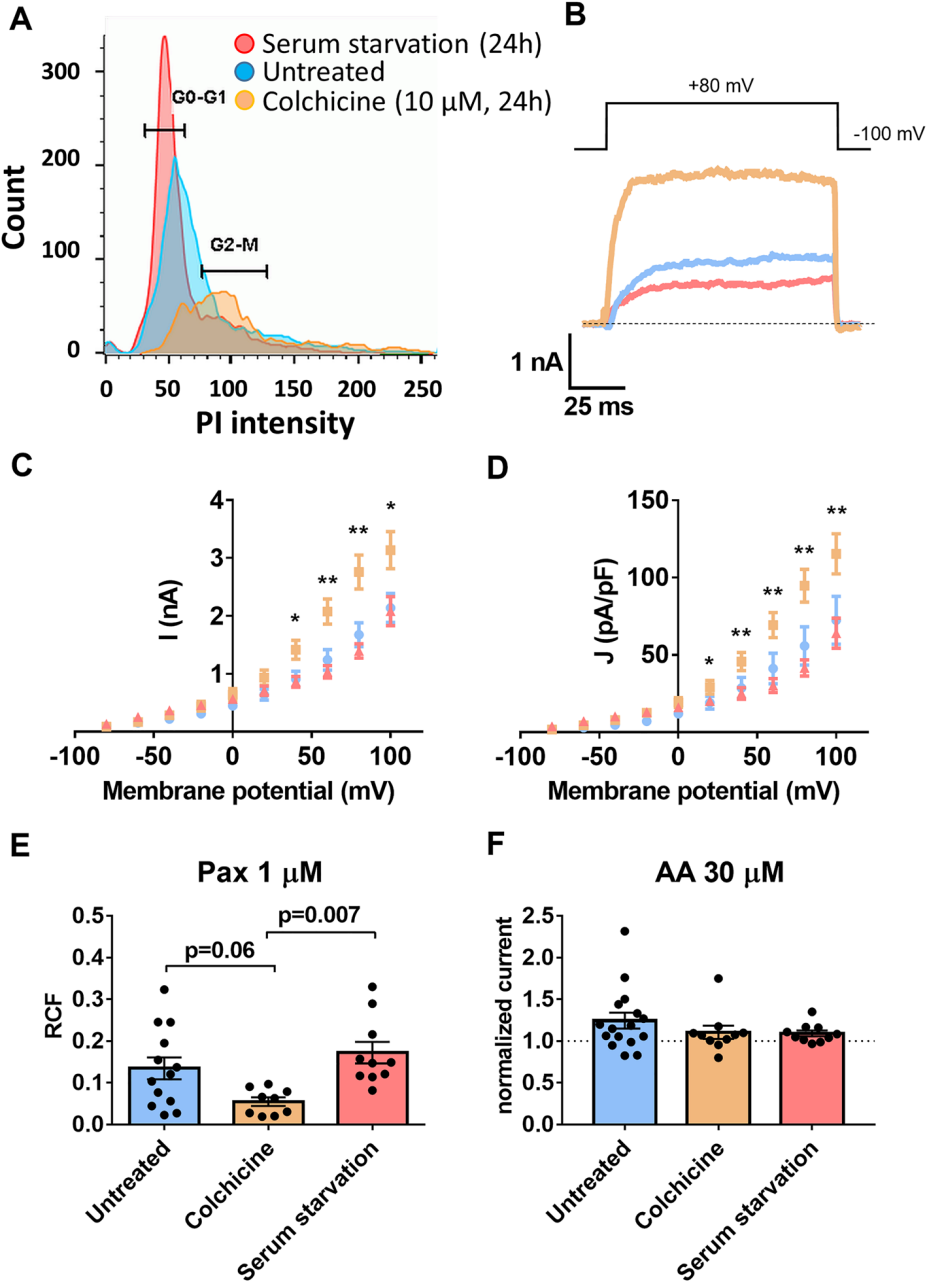
K_Ca_1.1 expression, but not the β3 subunit function shows cell cycle-dependence (**A**) Flow cytometry of colchicine treated (4 µg/ml, 24 h; in orange), starving (serum free DMEM, 24 h; in red) and untreated U-87 MG cells (in blue), where propidium iodide (PI) intensity is shown as a function of cell count. (**B**) Representative patch-clamp traces of the serum starved (red), colchicine-treated (orange) and untreated (blue) populations, respectively. The displayed currents were recorded at + 80 mV depolarizing pulse with 1 µM free Ca^2+^ in the pipette-filling solution. (**C**) Current amplitude (I, nA) as a function of membrane potential (mV) for control (untreated, blue, n = 20), for colchicine-treated (orange n = 19), and starving cells (n = 9). Whole-cell currents were obtained as in Fig. 1A (1 µM free Ca^2+^ in the pipette-filling solution). (**D**) Current density (J, pA/pF) as a function of membrane potential (mV) was calculated for n = 18 control (blue), n = 18 colchicine-treated (orange) and n = 7 starving cells (red) from the data in panel C. In panels C and D, asterisks indicate the significant difference between the colchicine treated and the untreated cells. (**E**) Effect of 1 µM paxilline (Pax) on the whole cell currents in untreated (n = 13), colchicine-treated (n = 9) and starving cells (n = 10). Black symbols indicate RCF values obtained in individual cells, RCF was calculated as in Fig. 1. (**F**) The effect of 30 µM AA on the normalized current in untreated (n = 16), colchicine-treated (n = 10), and starving cells (n = 10). Bars indicate the current amplitude measured with AA superfusion normalized to the current amplitude measured in control solution. Throughout the figure, data points, bars and error bars are mean ± SEM for the indicated number of experiments, *: *p* < 0.05; **: *p* < 0.01.

To investigate whether the K_Ca_1.1 β3 subunit is involved in downstream mechanisms of K_Ca_1.1 function, we studied the intracellular Ca^2+^ signaling evoked by the acetylcholine (ACh) analogue, carbachol^16,33^ (Fig. 5). Fig. 5A left panel shows that U-87 MG cells respond to the application of 10 µM carbachol with a marked increase in the cytosolic free Ca^2+^ concentration, as reported by the increased F_340_/F_380_ ratio. The Ca^2+^ response of the cells is inhibited by the simultaneous administration of carbachol and 1 µM paxilline (Fig. 5A, right panel). The heat maps in Fig. 6B highlight that approximately 50% of the cells in each population (59 of 120 cells in the control group, 49 of 91 cells in the carbachol + paxilline-treated group) respond to cholinergic stimulation by more than 20% increase in the F_340_/F_380_ ratio compared to their initial value. The statistical analysis (Fig. 5C) clearly indicates that paxilline (n=49 cells; peak F_340_/F_380_= 1.6 ± 0.06) inhibits the Ca^2+^ response of the U-87 MG cells to carbachol (n= 59 cells; peak F_340_/F_380_= 2.1 ± 0.07; *p*<0.0001). Interestingly, the peak of the carbachol-induced Ca^2+^ signal (Fig. 5D) at t = 220s is inhibited by β3 silencing (*KCNMB3* siRNA transfected, n=30; *p*=0.0004) similarly to silencing of the pore forming subunit of K_Ca_1.1 (*KCNMA1* siRNA transfected, n=51; *p*=0.025). The F_340_/F_380_, ratios at t = 220 s were 1.1 ± 0.06 (n=39) for the siGAPDH treatment, 0.8 ± 0.04 (n=51) for the siKCNMA1 (siKCNMA1), and 0.9 ± 0.04 for siKCNMB3 treatments (n=30), (Fig 5D). Based on our results we conclude that K_Ca_1.1 co-expressed with the β3 subunit mediates Ca^2+^-signaling in response to carbachol in U-87 MG cells.

As cytosolic Ca^2+^ fluctuates during the cell cycle and K^+^ channels are expressed in a cell cycle-dependent manner^34,35^, we aimed at testing whether β3 subunit-dependent modulation of the K_Ca_1.1 current is influenced by the cell cycle of glioblastoma cells. To this end, we synchronized U-87 MG cells in M phase using colchicine and in G_0_ phase using serum starvation (Fig. 6). Fig. 6A shows the flow cytometry data of the synchronized cells. The histograms and Supp. Table 3 show that 36 ± 3 % of the cells were in G_2_/M phase 24 h after a 10 µM colchicine treatment (N=3, n=3) as compared to 16 ± 1% in the untreated group (N=3, n=5). Upon serum starvation, a high percentage of cells reside in the G_0_/G_1_ phase (57 ± 3% for untreated, 78 ± 1% for serum starvation, N=3, n=5 and N=2, n=2, respectively) (Supp. Table 3). Interestingly, we observed a marked increase in the magnitude of the whole-cell currents in M phase synchronized cells as compared to control (non-synchronized, untreated) and G_0_ synchronized ones (Fig. 6B-C). The increase in the peak currents becomes evident at depolarizations to +40 mV or above (Mann-Whitney test, Fig. 6C). As cell volume and cell surface can also change during the cell cycle^36^, we also determined current density (J, see above, section “KCa1.1 is a prominent K+ channel in the plasma membrane of glioblastoma cells”). Similar to the peak currents, the current density was significantly larger in the M phase synchronized cells as compared to control and G_0_ phase synchronized ones at depolarized test potentials (above +20 mV, Mann-Whitney test, Fig. 6D). To ensure that the main component of the whole-cell currents remains K_Ca_1.1 in these cells, we applied paxilline (1 µM) to each synchronized and control cell population (Fig. 6E). We found a pronounced inhibition of the whole cell K^+^ current by paxilline in all cell cycle phases, especially in the M phase, the average RCF in colchicine- and serum starvation-treated cells were 0.05 ± 0.01 (n=9) and 0.17 ± 0.02 (n=10), respectively (*p*=0.007, Kruskal–Wallis test, Fig. 6E). This confirms that the major current component is K_Ca_1.1 in colchicine-synchronized cells. On the other hand, the increase in the whole-cell current induced by 30 µM AA was similar in all groups (current increase: 1.26 ± 0.09 (n=16), 1.1 ± 0.08 (n=10) and 1.09 ± 0.03 (n=10) for the untreated, colchicine- and serum starvation treated cells respectively, *p*=0.46, Kruskal-Wallis test, Fig. 6F). Together, these results indicate that K_Ca_1.1 function is increased after M phase synchronization, without alterations in the β3 subunit-dependent K^+^ current modulation.

## Discussion

In this study, we showed that the Ca^2+^- and voltage dependent K^+^ channel K_Ca_1.1 functions in the plasma membrane of patient-derived primary glioblastoma cells as well as the U-87 MG cell line in association with its auxiliary β3 subunit (Figs. 1, 2, 3 and 4). This is relevant for cellular Ca^2+^ signaling (Fig. 5) but not for cell cycle progression (Fig. 6). Even though K_Ca_1.1 is ubiquitously expressed in many tissues in the human body, auxiliary β subunits have a much more restricted tissue expression. Particularly, the β3 subunit is rarely found in healthy tissues^37^, and has only been described to date in the testes, pancreas and spleen. This may be relevant in diagnosis and/or therapy, especially since the increased expression of K_Ca_1.1 β3-encoding gene *KCNMB3* correlates with poor survival of GBM patients^10^ and increased β3 auxiliary subunit expression was described in fibroblast-like synoviocites in rheumatoid arthritis^9^. Also, an intriguing prospect of *KCNMB3* expression in high-grade gliomas^10^ is that based on our findings K_Ca_1.1 β3 can be a cell surface prognostic marker in GBM. Therefore, generating auxiliary subunit-specific probes is warranted in a future study.

Based on the following pieces of evidence we argue for the presence and functional activity of β3/K_Ca_1.1 α complexes on glioblastoma cells: (i) the transcript for β3 is highly expressed, its relative expression is similar to that of the α subunit in U-87 MG cells (Fig. 3A); (ii) the whole cell current was augmented by AA application (Fig. 2B), and this effect was diminished following silencing of the β3 auxiliary subunit (Fig. 3E); (iii) fast and incomplete inactivation of the whole cell current was recorded at high, 10 µM intracellular free Ca^2+^ concentration and depolarization to +180 mV (Fig. 4A-D); (iv) inward tail currents were recorded at −100 mV upon exposure of the cells to the reducing agent DTT (Fig. 4D-E). Moreover, sensitivity of the whole cell current to 23 nM IbTx is consistent with the pharmacology of the K_Ca_1.1/β3 complex^38^. Nevertheless, the findings listed in i.-iv*.* should be collectively interpreted and used as an argument for the presence of the β3 auxiliary subunit/K_Ca_1.1 α complex as many of these characteristics are shared with other β subunit/K_Ca_1.1 α complexes, as discussed below.

For example, the β1 subunit, prominently expressed in smooth muscle cells, prolongs activation kinetics of K_Ca_1.1 and has a well characterized pharmacological activation by bile acids^8,17,39,40^. Even though ionic currents of primary U-87 MG cells, and to a smaller extent in GBM, are activated by 75 μM lithocholic acid, two factors argue against the presence of K_Ca_1.1 β1 in glioblastoma cells. First, we could not detect K_Ca_1.1 β1 in RT-qPCR (Fig. 3A). In line with this, we observed similar pharmacological response in *KCNMB1*-silenced cells as in the scrambled siRNA-treated controls, as if the effect of LCA on the currents was oblivious to the presence or absence of the β1 subunit. Knowing that bile acids activate a multitude of other ion channels, e.g. bile acid sensitive ion channels (BASIC)^41^, it is much more likely that LCA acts on different ion channels than K_Ca_1.1 in glioblastoma cells. K_Ca_1.1 in complex with β1 is also quite resistant to IbTx inhibition (IC50~between ~65 nM^24^ to ~370 nM^38^, which is in contrast to our finding in Fig. 2D). Furthermore, it is known that β1 associated K_Ca_1.1 channels have instantaneous tail currents at negative membrane potentials^42^, and based on our recordings, we have only seen tail currents at negative membrane potentials when we treated the cells with 20 mM DTT (Fig. 4E and F).

Regarding the β2 subunit of K_Ca_1.1, we found that it is present in the cells on mRNA level as determined by RT-qPCR. β2 subunits are similar to β3 subunits in a manner that arachidonic acid increases the K_Ca_1.1 current if the channel is associated with them^20,29^. Moreover, *KCNMB2*-silenced U-87 MG cells show less AA-dependent response compared to control-silenced cells, indicating that KCa1.1 may be in complex with this auxiliary subunit. However, β2 subunits lead to a complete inactivation of K_Ca_1.1-mediated currents, even at low cytosolic Ca^2+^ concentration and modest depolarization to +100 mV, which was used to obtain data in Fig 2C ^22,23^. Complete inactivation of the current was rarely (n=2 out of n=51 primary GBM cells) observed in our settings, with most whole-cell currents showing no inactivation during 200 ms (Fig. 2C). We did not see the complete inactivation of the current either when the recording conditions were optimal to see the inactivation induced by the β2 subunits, i.e., when the free Ca^2+^ concentration in the pipette filling solution was 10 µM (Fig. 4). The lack of inactivation - characteristic to the presence of β2 - can be attributed to the variability in the stoichiometry between the different β subunits associated with to the α subunit of K_Ca_1.1: as four possible β subunits can simultaneously bind to one functional channel^43^, the ratio of different β subunits associated with the channel may become very important, as indicated previously^44,45^. In GBM, a biological consequence of altered subunit stoichiometry is easily possible: more β2 subunits linked to one K_Ca_1.1 channel would mean complete channel inactivation, thus less driving force for Ca^2+^ signals, whereas more β3 subunits would lead to a rapid but incomplete channel inactivation (as can be seen in Fig. 4) and a prolonged Ca^2+^ influx. Therefore, a thorough assessment is warranted in a further study to prove this concept in GBM.

The K_Ca_1.1 β4 subunit is known to be expressed in the central nervous system^37,46^. In transfected model cells, K_Ca_1.1 channels coupled to β4 subunit become resistant to inhibition by the peptide toxin IbTx^24,47^. Bicistronic expression experiments confirmed that when β4 is present in saturating stoichiometry the β4/K_Ca_1.1 complexes are insensitive to IbTx-mediated inhibition^38^. In contrast, we observed that IbTx inhibits whole-cell currents potently (Fig. 2D). Thus, it is likely that the β4 subunit is not associated with K_Ca_1.1 in glioblastoma. Lastly, K_Ca_1.1 gamma subunits, are unlikely in GBM cells: as K_Ca_1.1 channels associated with γ subunits already open at very negative membrane potentials of –150 mV^42^. In comparison, K_Ca_1.1 starts to conduct at a more positive membrane potential in primary GBM as well as in U-87 MG cells (Fig. 1A). In summary, besides the evident association of K_Ca_1.1 channels to β3 in the plasma membrane of GBM cells, it is likely that a minority of the channels may be coupled to β2.

To our knowledge, we are the first to describe that K_Ca_1.1, coupled to its auxiliary β3 subunit, modulates the Ca^2+^ signal upon ACh receptor stimulation (Fig. 5). The reduced Ca^2+^ signal in the presence of the K_Ca_1.1, inhibitor paxilline is consistent with a model where K_Ca_1.1-dependent membrane potential alterations provide the electrical driving force for Ca^2+^ entry^48^. Generally, the function of ancillary ion channel subunits is to fine-tune the expression and biophysical properties of the pore-forming (here K_Ca_1.1) α subunit^8,49,50^. It has been recently proposed that ACh-induced signals, in a Ca^2+^-dependent manner, induce matrix metalloprotease 9, which ultimately increases glioblastoma cell invasiveness^33^.

Interestingly, the K_Ca_1.1 channel together with the β3 subunit is also functional in fibroblast-like synoviocytes in rheumatoid arthritis^9^. One explanation for this can be that both glioblastoma and rheumatoid arthritis are accompanied by pronounced inflammation altering multiple parameters such as pH and the mechanical environment^51–53^. For example, it has been described that mechanosensitivity of K_Ca_1.1 is conferred by the auxiliary β1 subunit in vascular smooth muscle^54^. Whether alterations in the microenvironment indeed modify auxiliary subunit expression via a common mechanism in diseases involving inflammation remains to be elucidated.

## Conclusion

We found that K_Ca_1.1 channels are coupled primarily to the auxiliary β3 subunit in the cell membrane of glioblastoma and U-87 MG cells with functional consequences on Ca^2+^-signaling of GBM cells upon muscarinic acetylcholine receptor activation. The β3 subunit expression of GBM cells may allow either specific targeting of the tumor cells using β3 subunit specific inhibitors and/or allow diagnostic tools to be developed based on β3 subunit expression. In conclusion, we propose that the β3 subunit of the channel acts as a membrane-localized marker for glioblastoma cells to be exploited for diagnostic or therapeutic approaches.

## Materials and methods

### Glioblastoma cell isolation

Experiments on patient-derived GBM tissue samples were carried out under the approval of the Hungarian Research Ethical Committee (ETT-TUKEB, IV/186- 1 /2022/EKU). Diagnosis was established according to WHO criteria^55^ by a neuropathologist (T.H.). Informed consent was obtained for all human subjects involved in this study. Tumor samples were collected from anonymized adult patients during the surgical removal of the glioblastoma and transported for further processing in HBSS (Hank’s Balanced Salt Solution) on ice. Next, tissue samples were digested for 30 min in Collagenase type I (Sigma Aldrich, Burlington, MA, USA), and eventually homogenized using a tissue homogenizer and Pasteur pipettes, as modified from Souza et al.^56^. Lastly, single cell suspension was achieved using a 70 µm cell strainer (Corning, Corning, NY, USA). Single cells were left to adhere in DMEM + 10% FCS at 37 °C and 5% CO_2_ for 2 h, then washed 3× with PBS. Cells were incubated in DMEM medium including 10% FCS, 1% glutamate, 1% penicillin-streptomycin and 1% non-essential amino acids for a maximum of three passages. Glioblastoma cell purity was routinely assessed using GFAP immunocytochemistry^57^. Only those glioma populations were used for experiments, where >90% of cells showed clear GFAP positivity.

### Patch-clamp electrophysiology

Whole-cell currents of voltage-clamped cells were recorded by manual patch-clamp electrophysiology according to standard protocols using an Axopatch 200B amplifier connected to a computer and digitized with Digidata 1550B (Molecular Devices, CA, USA). Pipettes were pulled from GC 150F-15 borosilicate glass capillaries (Harvard Apparatus, MA, USA) in five stages with 4–10 MΩ resistance. Immediately before the measurement, the cells were maintained in the recording petri dish in a bath solution consisting of 145 mM Na-aspartate, 5 mM KCl, 1 mM MgCl_2_, 2.5 mM CaCl_2_, 5.5 mM glucose, and 10 mM HEPES, pH = 7.4, titrated with NaOH. The composition of the solution used in the patch pipette (internal solution) was either Ca^2+^-free (composition: 140 mM KF, 2 mM MgCl_2_, 1 mM CaCl_2_, 10 mM HEPES, and 11 mM EGTA, pH = 7.22, titrated with KOH) or contained 1–10 µM free Ca^2+^ (145 mM K-aspartate, 10 mM EGTA, 10 mM HEPES, 2 mM MgCl_2_, and 8.5 mM CaCl_2_, pH = 7.2, titrated with Tris). K_Ca_1.1 channel modulators (lithocholic acid, arachidonic acid, iberiotoxin, paxilline and dithiothreitol(DTT); Sigma Aldrich, Burlington, MA, USA) were freshly diluted to the desired concentration (75 µM, 30 µM, 23 nM, 1 µM and 20 mM respectively) in the bath solution. The solvents of the stock (DMSO or ethanol) were diluted to ≤0.1% V/V in the bath solution. Solution exchange was achieved by using a gravity-flow perfusion system (flow rate: 2 ml/min) with continuous excess fluid removal. For the biophysical characterization of the K_Ca_1.1 currents, the cells were depolarized from a holding potential of −100 mV to +100 mV in +20 mV increments. We used a 100 or 200 ms depolarization protocol from −100 mV to +100 mV for testing of K_Ca_1.1 channel modulators and 20 ms-long test pulses to +180 mV from −180 mV holding potential to study inactivation, and measured the instantaneous currents at −100 mV. All experiments were carried out at room temperature. Voltage-clamp data were acquired with pClamp10 (Molecular Devices, CA, USA). In general, currents were low-pass-filtered using the built-in analog four-pole Bessel filters of the amplifiers and sampled at 20 and 50 kHz. Whole-cell current traces were digitally filtered (five-point boxcar smoothing) before analysis. Clampfit 10.7 (Molecular Devices, CA, USA) and GraphPad Prism 7 (GraphPad, CA, USA) were used for data display and analysis.

### Immunocytochemistry

We followed standard immunofluorescence protocol as described in^13^ for fluorescent detection of K_Ca_1.1 and the glial fibrillic acidic protein (GFAP). Briefly, primary patient-derived GBM cells were plated onto coverslips after the first passage. After overnight adhesion, cells were washed, then fixed (4% paraformaldehyde + 0.1% Triton-X-100 in PBS) at room temperature for 20 min. After washing and blocking (10% goat serum in PBS) at room temperature for 1 h, cells were labeled with antibodies against K_Ca_1.1α (1:200 dilution of AB5228; rabbit polyclonal, Merck Millipore, Darmstadt, Germany) and/or GFAP (1:500 dilution of G3893; mouse monoclonal, Sigma Aldrich, Burlington, MA, USA) at 4 °C overnight. After washing, fluorescent secondary antibodies against mouse (405324, Alexa-555 rabbit polyclonal, Biolegend, CA, USA) and rabbit (A-21244, Alexa-647 goat polyclonal, Invitrogen, MA, USA) were applied at 1:1000 dilution at 4 °C for 2 h. Lastly, after washing coverslips were mounted onto slides using DAKO mounting medium (Agilent, Santa Clara, CA, USA). Acquisition and qualitative assessment of the stainings were performed at 40× magnification using a confocal microscope (Olympos FV1000). Cells were labelled GFAP positive when the intracellular staining had a typical filamentary phenotype, and K_Ca_1.1 staining was considered positive when it had a punctate membrane staining pattern typical of ion channels^13,14^.

### RNA isolation and RT-qPCR

RNA was isolated from primary GBM cells after 24 h of culture as well as from cultured U-87 MG cells using TRIzol^TM^ (Life Technologies, Carlsbad, CA, USA) following manufacturer’s instructions. cDNA was generated using the Superscript III™ Reverse Transcriptase kit (Invitrogen, Waltham, MA, USA) with 2  μg of RNA per reaction. RT-PCR was performed using a QuantStudio 3 cycler with PowerUp™ SYBR™ Green Master Mix (Applied Biosystems®/Thermo Fisher Scientific, Waltham, MA, USA), according to manufacturer´s instructions. Data was evaluated using the QuantStudio Design and Analysis software (Applied Biosystems®/Thermo Fisher Scientific, Waltham, MA, USA). Primer sequences are listed in Supp. Tables 1 and 2 for of U-87 MG cells primary patient-derived GBM cells, respectively.

### siRNA application

The U-87 MG cells were transfected with siRNA according to manufacturer´s instructions by Dharmacon™ (Horizon Discovery, Lafayette, CO, USA). Briefly, cells were transfected in Gibco® Opti-MEM™ medium (Thermo Fisher Scientific, Waltham, MA, USA), containing 2 µl/ml DharmaFECT™ (Horizon Discovery, Lafayette, CO, USA) and 5 µg/ml scrambled siRNA (AccuTarget™ Negative Contol siRNA, Bioneer, Daejeon, South Korea); or 5 µg/ml of a mixture (SMARTpool) of ON-TARGETplus siRNA against either GAPDH (siGAPDH) KCNMA1 (siKCNMA1), KCNMB1 (siKCNMB1), KCNMB2 (siKCNMB2) or KCNMB3 (siKCNMB3) at 37 °C and 5% CO_2_ for 24 to 48 h before patch-clamp and Western blot analysis. Gene silencing was validated using Western Blot (Fig. 3B).

### Western blot

Protein from U-87 MG cells was isolated using TRIS lysis buffer (25 mM mercaptoethanol, 1 µl/ml Tween 20, 10 µl/ml protease inhibitor, 50 mM TRIS base, pH=7.5) and sonication for 30 s. 120 µg of denatured protein sample were loaded into each well of the 12% ProSieve 50 (Lonza, ME, USA) modified acrylamide gel for electrophoresis (80 mV for 20 min, then 120 mV for 90 min), followed by transfer onto PVDF membranes (100 mV for 90 min). After blocking (5% skim milk powder in 10 mM TRIS-buffered saline) at 4 °C for 1 h, the blocked membranes were incubated at 4 °C overnight with 1:1000-fold dilutions of primary antibodies against the K_Ca_1.1 β subunits or actin: anti-KCNMB1 (nb300-535, rabbit polyclonal, Novus Biologicals, CO, USA), anti-KCNMB2 (MA5-27646, mouse monoclonal, Thermo Fisher Scientific, Waltham, MA, USA), anti-KCNMB3 (ab137041, rabbit monoclonal, Abcam, Cambridge, UK), anti-actin (a2066, rabbit polyclonal, Sigma-Aldrich, MO, USA). After washing three times, blots were incubated 1:10 000-fold diluted secondary anti-mouse (#7076, Cell Signaling Technology, MA, USA) or anti-rabbit antibodies (#7074, Cell Signaling Technology, MA, USA) at 4 °C for 2 h. Blot chemiluminescence was detected using a commercial detection system (Chemidoc XRS, Bio-Rad, Hercules, CA, USA).

### Cell synchronization and cell-cycle analysis

For metaphase cell synchronization, U-87 MG cells were incubated with 4 µg/ml colchicine (Sigma Aldrich, Burlington, MA, USA)-containing medium at 37 °C and 5% CO_2_ for 24 h. To measure the efficacy of synchronization, colchicine-synchronized as well as the untreated U-87 MG cells were fixed and permeabilized with 80% ethanol at room temperature for 20 min and stained with 2 µg/ml propidium-iodide (PI) at room temperature for 10 min for flow cytometry measurements^58^. The data were acquired with BD FacsAria III Cell Sorter (BD Biosciences, NJ, USA). 561 nm excitation laser and 610/20 nm emission filter with 600 nm long-pass dichroic mirrors were used for event detection. Data was subsequently evaluated with Flowjo V10 software (BD, Franklin Lakes, NJ, USA).

### Intracellular Ca^2+^ measurements

U-87 MG cells were loaded with 3 µM Fura-2-AM (Invitrogen, Waltham, MA, USA)-containing HEPES-buffered Ringer`s solution with glucose (140 mM NaCl, 5.4 mM KCl, 1.2 mM CaCl_2_, 0.8 mM MgCl_2_, 5.5 mM D-glucose, and 10 mM HEPES, titrated to pH = 7.4, titrated with NaOH) for 20 min at 37 °C. Next, cells were washed twice with fresh Ringer`s solution and were then visualized using an imaging setup composed of a Zeiss AxioVert 100 inverted fluorescence microscope (Zeiss, Oberkochen, Germany), a high-speed shutter, a polychromator (Visitron Systems, Puchheim, Germany) and a 37 °C acquisition cabin. Fura-2 excitation wavelengths were 340 nm and 380 nm, corresponding to the Ca^2+^-loaded and Ca^2+^-free excitation optima, respectively. Fluorescence emission was recorded at a wavelength of 510 nm. The ratio of the fluorescence intensities emitted upon 340 nm and 380 nm excitation (F_340_/F_380_) is directly proportional to the intracellular Ca^2+^ concentrations and was used in this study to report the cytosolic free Ca^2+^ concentration^59^. The cells were kept at 37 °C during the whole measurement. During the acquisition, cells were initially superfused with the control solution (0.1% DMSO in Ringer´s solution) for 2 min, followed by 5 min with either only 10 µM acetylcholine (Ach)-receptor agonist carbachol (carbamylcholine chloride; Sigma Aldrich, Burlington, MA, USA)-containing Ringer´s solution to elicit a Ca^2+^-signal (previously described by^16,33^), or with 10 µM carbachol + 1 µM paxilline containing Ringer´s solution to simultaneously inhibit K_Ca_1.1. Ratios were evaluated with the Visiview 3.0 software (Visitron Systems, Puchheim, Germany), and ultimately, individual F_340_/F_380_-curves were visualized using R^60^.

### Statistical analysis

Data are presented as mean ± SEM. Statistical analysis was carried out using GraphPad Prism 7. Following a D’Agostino-Pearson normality test, unpaired Student's *t* tests or one-way ANOVA were performed with Tukey’s post hoc test, in other cases Mann-Whitney or Kruskal–Wallis tests were used. To assess the effects of the channel modulators we performed Wilcoxon signed-rank tests. Statistical significance was assumed when *p* < 0.05.

## Supplementary Information

Supplementary Information.

## Data Availability

The data that supports the findings is available upon request from the corresponding author panyi@med.unideb.hu.

## Abbreviations

GBM: Glioblastoma multiforme
PAX: Paxilline
LCA: Lithocholic acid
AA: Arachidonic acid
Ibtx: Iberiotoxin
gBK: Glioma BK
GFAP: Glial fibrillary acidic protein
RCF: Remaining current fraction
PI: Propidium iodide
Ach: Acethylcholine
