# Supplementary material for: Mapping the functional expression of auxiliary subunits of KCa1.1 in glioblastoma
Source: Sci Rep. 2022 Dec 20;12:22023. doi: 10.1038/s41598-022-26196-w (PMC9768140; doi:10.1038/s41598-022-26196-w)
Supplement: Supplementary file 1 — Supplementary Information. [file 41598_2022_26196_MOESM1_ESM.docx]

**Supplementary information**

**Mapping the functional expression of auxiliary subunits of K_Ca_1.1 in glioblastoma**

## Adam Feher1^#^, Zoltán Petho˝1,2^#^, Tibor G. Szanto1, Álmos Klekner3, Gabor Tajti1, Gyula Batta4, Tibor Hortobágyi^5,6^, Zoltan Varga1, Albrecht Schwab2 and Gyorgy Panyi1*

*1Department of Biophysics and Cell Biology, Faculty of Medicine, University Debrecen, Debrecen, Hungary*

*2Institute of Physiology II, University Münster, Münster, Germany*

*3Department of Neurosurgery, Faculty of Medicine, University Debrecen, Debrecen, Hungary*

*4Department of Genetics and Applied Microbiology, University Debrecen, Debrecen,*

*Hungary*

*^5^Institute of Pathology, Faculty of Medicine, University of Szeged, Szeged, Hungary.*

*^6^ELKH-DE Cerebrovascular and Neurodegenerative Research Group, Department of Neurology, Faculty of Medicine, University Debrecen, Debrecen, Hungary*

*Corresponding author, Tel.: +36 52 411 717 / 65617, E-mail: [panyi@med.unideb.hu](mailto:panyi@med.unideb.hu)

# These authors contributed equally to the manuscript

Supplementary Methods

1. RT-PCR and gel electrophoresis

The cDNA of primary GBM patients was obtained as described in section 2.4 and was used as a template for PCR using Biozym Red HS Taq Master Mix (Biozym, Hessisch Oldendorf, Germany), with the primers detailed in Supp. Table 2. PCR conditions were following: 40 cycles of denaturation at 95 °C for 30 s, annealing at 55 °C for 45 s, and extension at 72 °C for 60 s in an Eppendorf[®](https://www.google.com/url?sa=i&url=https%3A%2F%2Fwww.sigmaaldrich.com%2Fcatalog%2Fproduct%2Fsigma%2Fz316091&psig=AOvVaw301T-qa9USydlTbgg5Sn81&ust=1644131562144000&source=images&cd=vfe&ved=2ahUKEwjZwom_gej1AhUFkRoKHXMlDX4Qr4kDegUIARDDAQ) Mastercycler (Eppendorf, Hamburg, Germany). PCR products were separated by 1.5% agarose gel electrophoresis and images were acquired under UV light. Product band intensities were evaluated by ImageJ.

Supplementary Figures

**Supp. Figure 1**: Primary patient-derived GBM cells express β subunits of K_Ca_1.1.

Band intensities of KCNMA1 (K_Ca_1.1) and the respective beta-subunit encoding genes were normalized to the intensity of GAPDH after gel electrophoresis of RT-PCR products (N=3).

**
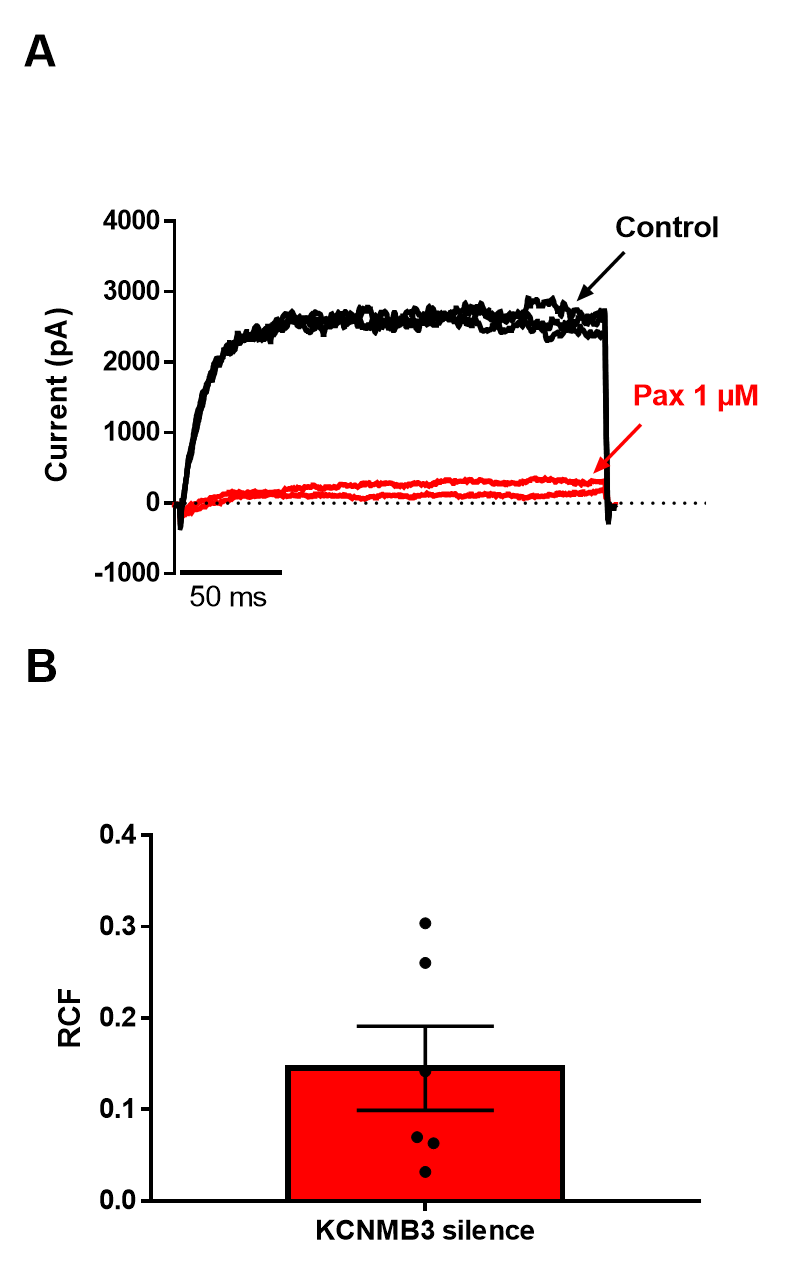
**

**Supp. Figure 2:** The effect of paxilline on KCNMB3 silenced U-87 MG cells

The KCNMB3 gene encoding the beta3 subunit was silenced in U-87 MG cells using siRNA as described in the Materials and Methods section. **A)** Representative measurement of the KCa1.1 current using whole-cell patch clamp in a KCNMB3 silenced U-87 MG cell. Currents were evoked by repeated depolarizations to +100 mV from a holding potential of -100 mV every 15 s in control extracellular solution (black) and upon reaching equilibrium block in the presence of 1 µM paxilline (red). The pipette filling solution contained 1 µM Ca^2+^ concentration. **B)** Column chart of the remaining current fraction (RCF = I/I_0_, where I_0_ and I are the peak currents in the absence and in the presence of the inhibitor, respectively) upon 1 µM paxilline administration. Black dots represent individual measurements, horizontal line and error bars indicate mean ± SEM.

**
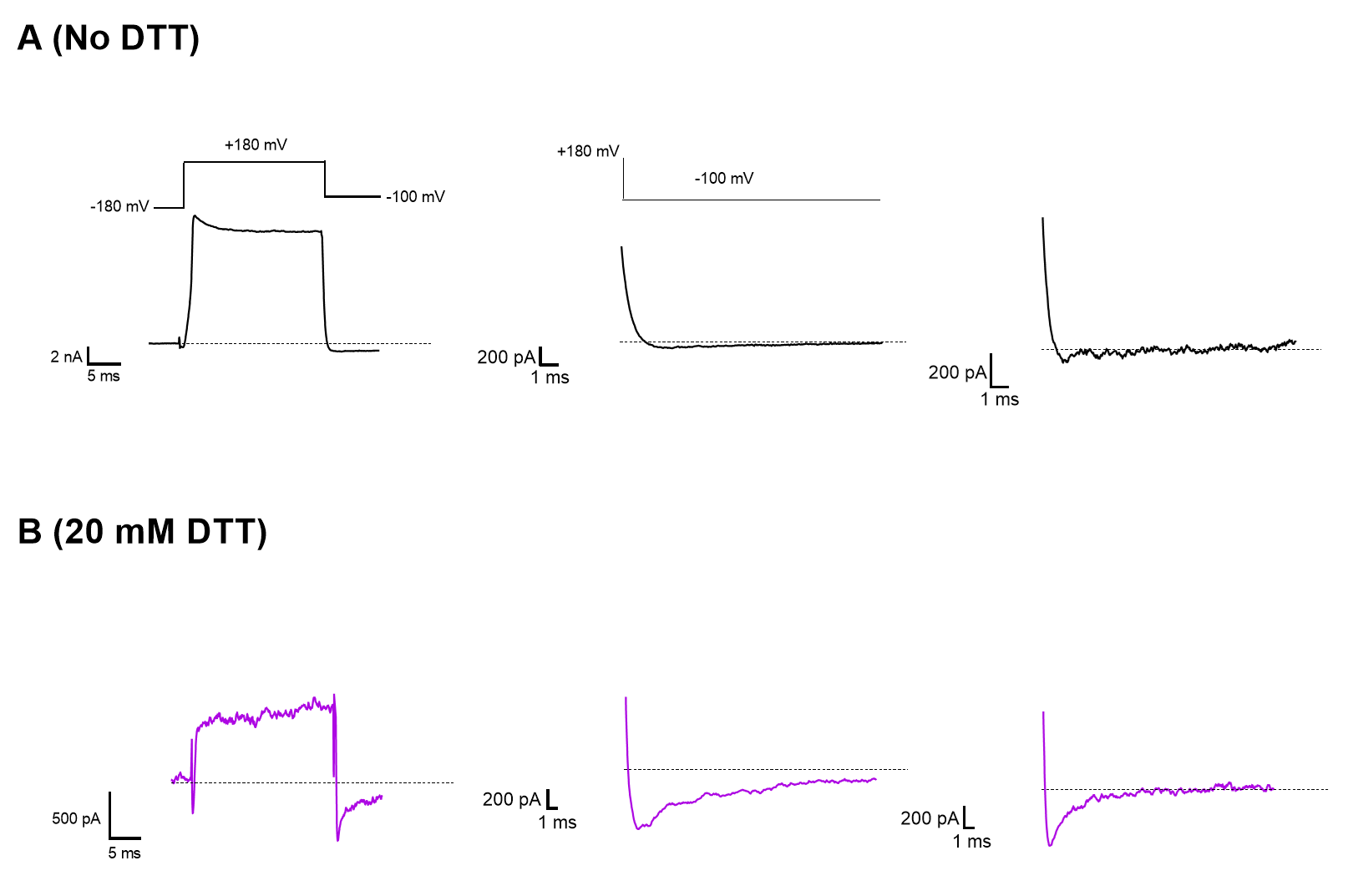
**

**Supp. Figure 3**: DTT treatment results in the appearance of the instantaneous current at -100 mV

This figure shows the 3-3 individual measurements displayed in the scatter plot in Fig. 4F. Whole-cell currents were recorded in U-87 MG cells using whole-cell patch clamp. The pulse protocol is shown in the left panel of A. The pipette filling solution contained 10 µM free Ca^2+^ concentration, other conditions are explained in Fig. 4 of the main text. **A.)** The left panel shows the current at full scale, the middle and right panels display the currents upon stepping the membrane potential to -100 mV from +180 mV to evoke the tail currents at an extended time scale. **B.)** The same set of experiment as in A except the cell was incubated with 20 mM DTT for 20 min.

Supplementary Tables

Supp. Table 1. Primer sequences used for RT-qPCR of U-87 MG cells

| Primers used for qPCR of U-87 MG cells (as described in Ref [42]) | | | |
| --- | --- | --- | --- |
| Gene Name | RefSeq | Forward/reverse | Sequence |
| KCNMA1 | NM_001014797 | FW | GCT CAA GTA CCT GTG GAC CG |
|  |  | Rev | CTG GTT TGA GAG TGC CAT CC |
| KCNMB1 | NM_004137.4 | FW | CTG TAC CAC ACG GAG GAC ACT |
|  |  | Rev | AGG TGC GGA GAA GTA GA |
| KCNMB2a | NM_181361.3 | FW | TGA CAA CTA CCG GAG GTC TT |
|  |  | Rev | GGA GGT CAT GGT CCC TGA TT |
| KCNMB2b | NM_001278911.2 | FW | GAG AAA GAG CAA CAA AGC GG |
|  |  | Rev | TTA GCA AAT CCC AGA CAT TGC |
| KCNMB3a | NM_171828.2 | FW | AAA TCA CAC TTC AGG GCA GC |
|  |  | Rev | GCA CAT CTA GTG GGT CTC CA |
| KCNMB3b | NM_171829.2 | FW | TCT GAG TGT GAG GGG CTC TT |
|  |  | Rev | GCA CAT CTA GTG GGT CTC CA |
| KCNMB3c | NM_171830.1 | FW | TCA CGA GAC ACA GGT AGG CAG CAA |
|  |  | Rev | TGG CAA ACC CCA GCA TCA CGG |
| KCNMB3d | NM_014407.3 | FW | GCT CAC GAG ACA CAG GAC AGC CTT |
|  |  | Rev | TGG CAA ACC CCA GCA TCA CGG |

Supp. Table 2. Primer sequences used for RT-PCR of primary patient-derived GBM cells

| Gene Name | RefSeq | Forward/reverse | Sequence |
| --- | --- | --- | --- |
| KCNMA1 | NM_001014797 | FW | CGG TGA CCA TGG AGG T |
|  |  | Rev | AGA GAA GGA AGA ACA CGT TG |
| KCNMB1 (β1) | NM_004137.4 | FW | CAC CTA CTA CAT CCT GGT CAC |
|  |  | Rev | TGG ATG GAT GGC TCT ACT TC |
| KCNMB2 (β2) | NM_181361.3 | FW | GTC ACT CCT ATT GTC CTT CC |
|  |  | Rev | CAA GGT GCA TTG AGA CTC TT |
| KCNMB3a (β3a) | NM_171828.2 | FW | TGT CTA AGC TTC ACC TTT CTT G |
|  |  | Rev | CAA ACA CCT GAA GAC ACG G |
| KCNMB3b (β3b) | NM_171829.2 | FW | TGT CAG AAC ATC CAA GTC AG |
|  |  | Rev | CAG GTC GAT TCT TCT CTC TG |
| KCNMB3c (β3c) | NM_171830.1 | FW | CAT TTC TTA GGC TTG TTT GCT |
|  |  | Rev | GGT CGA TTC TTC TCT CTG AA |
| KCNMB3d (β3d) | NM_014407.3 | FW | GCA TGA GGC AAC ATT TCT TA |
|  |  | Rev | AGC CAG TCG TCC ATG ATA |
| KCNMB4 (β4) | NM_014505.6 | FW | TCG GCT TGT TTC TCA TCA TCT |
|  |  | Rev | GAA GCA CAT CAT CTG GTC TTT |
| GAPDH | NM_002046.5 | FW | TGA AGG TCG GAG TCA ACG GATT |
|  |  | Rev | CAT GTG GGC CAT GAG GTC CAC CAC |

Supp. Table 3. Cell cycle analysis of U-87 MG cells following synchronization

| Unsynchronized | % Cells (mean ± SEM) |
| --- | --- |
| G_0_/G_1_ | 57.3 ± 3.2 |
| S | 26.6 ± 2.4 |
| G_2_/M | 16.1 ± 0.9 |
|  |  |
| Serum Starvation | % Cells (mean ± SEM) |
| G_0_/G_1_ | 77.8 ± 1.3 |
| S | 12.2 ± 0.8 |
| G_2_/M | 10 ± 0.6 |
|  |  |
| Colchicine | % Cells (mean ± SEM) |
| G_0_/G_1_ | 33.9 ± 6.3 |
| S | 31 ± 2.3 |
| G_2_/M | 35.1 ± 4 |
|  |  |
| Paxilline | % Cells (mean ± SEM) |
| G_0_/G_1_ | 54.4 ± 3.1 |
| S | 30 ± 2.2 |
| G_2_/M | 15.6 ± 0.9 |

Western blot images:

(Fig. 3B top box)


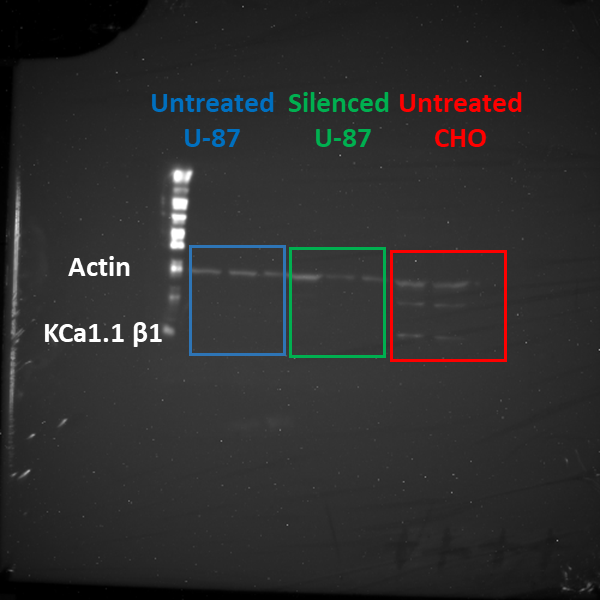


(Fig. 3B second box)


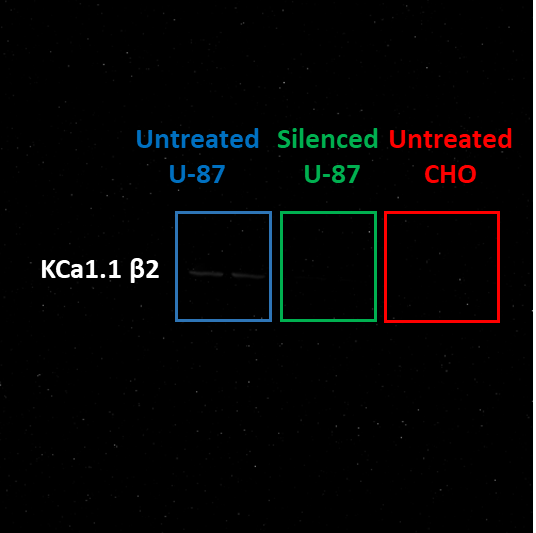


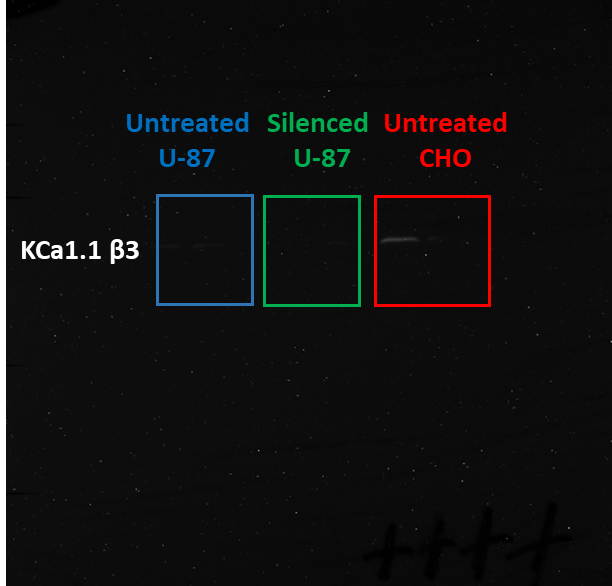
(Fig. 3B third box)
